# Multifrequency excitation of a clamped–clamped microbeam: Analytical and experimental investigation

**DOI:** 10.1038/micronano.2016.2

**Published:** 2016-03-14

**Authors:** Nizar Jaber, Abdallah Ramini, Mohammad I. Younis

**Affiliations:** 1 Physical Science and Engineering Division, King Abdullah University of Science and Technology, Thuwal 23955-6900, Saudi Arabia; 2 Department of Mechanical Engineering, State University of New York at Binghamton, Binghamton, NY 13902, USA

**Keywords:** multifrequency, nonlinear dynamics, wideband resonator, electrostatic, combination resonances

## Abstract

Using partial electrodes and a multifrequency electrical source, we present a large-bandwidth, large-amplitude clamped–clamped microbeam resonator excited near the higher order modes of vibration. We analytically and experimentally investigate the nonlinear dynamics of the microbeam under a two-source harmonic excitation. The first-frequency source is swept around the first three modes of vibration, whereas the second source frequency remains fixed. New additive and subtractive resonances are demonstrated. We illustrated that by properly tuning the frequency and amplitude of the excitation force, the frequency bandwidth of the resonator is controlled. The microbeam is fabricated using polyimide as a structural layer coated with nickel from the top and chromium and gold layers from the bottom. Using the Galerkin method, a reduced order model is derived to simulate the static and dynamic response of the device. A good agreement between the theoretical and experimental data are reported.

## Introduction

Microelectromechanical systems (MEMS) resonators are the primary building blocks of several MEMS sensors and actuators that are used in a variety of applications, such as toxic gas sensors^1^, mass and biological sensors^2–5^, temperature sensors^6^, force and acceleration sensors^7^, and earthquake actuated switches^8^. MEMS resonators can be based on thin-film surface micromachining, yielding compliant resonating structures, or bulk micromachining, for example, in the case of bulk resonators. These are primarily based on the wave propagation within the bulk structure. This article addresses the first category, that is, primarily clamped–clamped microbeam resonators.

MEMS resonators are excited using different types of forces, such as piezoelectric^9^, electromagnetic^10^, thermal^11^, and electrostatic^8,12^. The electrostatic excitation of resonators is the most commonly used method because of its simplicity and availability^12^. However, electrostatic forces are inherently nonlinear, thus adding complexity to the dynamics of these resonators, especially when they undergo large motions. The nonlinear dynamics of electrostatically actuated resonators have been thoroughly studied over the past two decades^12–19^.

There has been increasing interest in obtaining resonant sensors with large-frequency bands, especially with a high-quality factor range and near higher order modes of vibrations, where a high sensitivity of detection is demanded^1,2^. A few of the approaches that have been investigated to improve the vibration of resonators and increase their frequency band width are through parametric excitation^16^, secondary resonance^20^, slightly buckled resonators^21^, and multifrequency excitation^22^. Challa *et al.*^23^ designed and tested a device with tunable resonant frequency for energy harvesting applications. The resonant frequency band was increased up to ±20% of the original resonant frequency using a permanent magnet. The effects of the double potential well systems on the resonant frequency band and their application in energy harvesting applications are reviewed in Ref. 24. Recent studies on a carbon nanotube-based nano-resonator for mass detection applications proved that the resonator bandwidth is directly proportional to the forcing amplitude^25^.

Recent studies have highlighted the interesting dynamics of mixed frequency excitation and their applications as sensors and actuators. The mixed frequency excitation of a micromirror has been studied extensively in Ref. 22, where it is proposed as a method to improve the bandwidth in resonators. Erbe *et al.*^26^ demonstrated using the nonlinear response of a strongly driven nanoelectromechanical system resonator as a mechanical mixer in the radiofrequency regime. They used a magnetic field at an extremely low temperature (4.2 K) to excite a clamped–clamped microbeam using two AC signals of a frequency that were extremely close to each other and to the fundamental natural frequency of the beam. They determined that by exceeding a certain threshold of the amplitude excitation, higher order harmonics appeared. By increasing the excitation amplitude further, a multitude of satellite peaks with limited bandwidth occurred, thus allowing effective signal filtering. These results were verified by applying a perturbation theory on the Duffing equation with cubic nonlinearity and numerically integrating the Duffing equation and calculating the power spectrum of the response. They determined from both analysis and experiment that the cubic nonlinearity was responsible for generating the frequency peaks. A parametrically and harmonically excited microring gyroscope was investigated at two different frequencies in Ref. 27. The method proposed in the present study increases the signal to noise ratio and improves the gyroscope performance. Liu *et al.*^28^ fabricated and characterized an electromagnetic energy harvester, which harvested energy at three different modes of vibration. Moreover, the method of multifrequency excitation was implemented to perform mechanical logic operation, where each frequency carried a different bit of information^29^. The mixed frequency is used for an atomic force microscope resonator to generate high-resolution imaging and extract the surface properties^30^.

Motivated by the interesting dynamics and the wide range of applications of a large bandwidth resonator excited near the higher order modes of vibration, the objective of this article is to excite higher order modes of vibrations combined with multifrequency excitation to broaden the frequency bandwidth around the excited modes. The behavior of clamped–clamped microbeams excited by a multifrequency electrical source has been investigated experimentally and analytically.

## Materials and methods

### Fabrication

**Figure 1:**
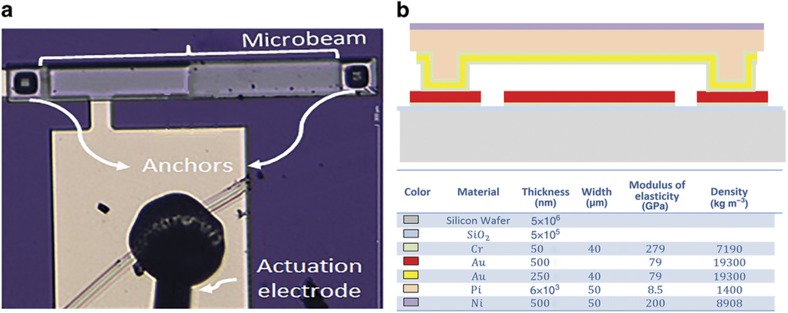
Fabrication and composition of the clamped–clamped microbeam resonator. (**a**) Top view of the fabricated microbeam with half of the lower electrode configuration and the actuation pad; and (**b**) cross sectional view of the fabricated microbeam depicting the different layer thicknesses and material properties.

The clamped–clamped microbeam resonator, as depicted in Figure 1a, is fabricated using the in-house process developed in Refs. 31,32. The microbeam consists of a 6-μm polyimide structural layer coated with a 500-nm nickel layer from the top and 50 nm chrome, 250 nm gold, and 50 nm chrome layers from the bottom. The nickel layer acts as a hard mask to protect the microbeam during the reactive ion etching process and defines the length and width of the beam. The lower electrode is placed directly underneath the microbeams and is composed of gold and chrome layers. The lower electrode provides the electrical actuation force to the resonator. The two electrodes are separated by a 2-μm air gap. When the two electrodes are connected to an external excitation voltage, the resonator vibrates in the out-of-plane direction. Figure 1b illustrates the various layers of the fabricated resonator.

### Problem formulation

**Figure 2:**
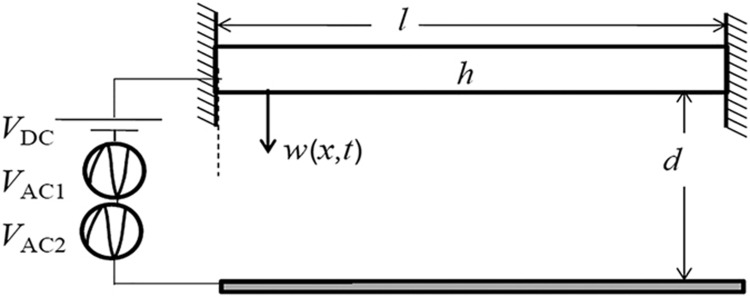
Schematic of the clamped–clamped resonator excited by a multifrequency electrical source.

We investigate the governing equation for a clamped–clamped microbeam depicted in Figure 2, which is electrostatically actuated by two AC harmonic loads *V*_AC1_ and *V*_AC2_ of frequencies *Ω*_1_ and *Ω*_2_, respectively, and superimposed onto a DC load *V*_DC_. The equation of motion governing the dynamics of the microbeam can be written as follows:

(1)EI∂4w∂x4+ρA∂2w∂t2+c∂w∂t=∂2w∂x2(N+EA2l∫01(∂w∂x)2dx)+εb[VDC+VAC1cos(Ω1t)+VAC2cos(Ω2t)2]2(d−w)2

where *E* is the modulus of elasticity; *I* is the microbeam moment of inertia; *c* is the damping coefficient; *A* is the cross sectional area; *ρ* is the density; *ε* is the air permittivity; *d* is the air-gap thickness; *t* is time; *x* is the position along the beam; *N* is the axial force; *b* is the beam width; and *w* is the microbeam deflection. The boundary conditions of the clamped–clamped microbeam can be given as follows:

(2)w(0,t)=0∂w∂x(0,t)=0w(l,t)=0∂w∂x(l,t)=0

Next, we non-dimensionalize the equation of motion and its boundary conditions for convenience. Accordingly, the non-dimensional variables (denoted by hats) can be introduced as follows:

(3)wˆ=wd,xˆ=xl,tˆ=tT

where *T* is a time scale that can be defined as follows:

(4)T=ρbhl4EI

By substituting Equations (3) and (4) into Equations (1) and (2) and dropping the hats from the non-dimensional variables for convenience, the non-dimensional equation can be derived as follows:

(5)∂4w∂x4+∂2w∂x2+cnon∂w∂t=∂2w∂x2(Nnon+α1∫01(∂w∂x)2dx)+α2[VDC+VAC1cos(Ω1t)+VAC2cos(Ω2t)]2(1−w)2

where the normalized boundary conditions are

(6)w(0,t)=0∂w∂x(0,t)=0w(1,t)=0∂w∂x(1,t)=0

The parameters in Equation (5) can be defined as follows:

(7)cnon=12cl4ETbh3;α1=6(dh)2;α2=6εl4Eh3d3;Nnon=12Nl2Ebh3

To calculate the beam response, we solve the normalized microbeam equation, Equation (5), in conjunction with its boundary conditions, Equation (6), using the Galerkin method^12^. This method reduces the partial differential equation into a set of coupled second order differential equations. The microbeam deflection can be approximated as follows:

(8)w(x,t)=∑i=1nϕi(x)ui(t)

where *ϕ*_*i*_(*x*) is selected to be the *i*^th^ undamped, unforced and linear orthonormal clamped–clamped beam mode shape; *u*_*i*_(*t*) is the *i*^th^ modal coordinate; and *n* is the number of assumed modes. To determine the mode shape functions *ϕ*(*x*), we can solve the eigenvalue problem as follows:

(9)ϕ(4)(x)−Nnonϕ(2)(x)−ωnon2ϕ(x)=0

where *ω*_non_ is the eigenfrequency. Both sides of Equation (5) are multiplied by (1−*w*)^2^ to simplify the spatial integration of the forcing term^12^. Then, we substitute Equation (8) into Equation (5) and multiply the outcome by the mode shape *ϕ*_*i*_(*x*). Next, we can integrate the resulting equation from 0–1 over the spatial domain as follows:

(10)∫01ϕj(1−∑l=1nulϕl)2(∑i=1nuiωnon,i2ϕi+∑i=1nüiϕi)dx+cnon∫01ϕj(1−∑l=1nulϕl)2(∑i=1nu˙iϕi)dx−α1∫01ϕj(1−∑l=1nulϕl)2{∑i=1nuiϕ′′i∫01(∑k=1nukϕ′k)2}dx=α2[VDC+VAC1cos(Ω1t)+VAC2cos(Ω2t)]2∫01ϕjdx

Evaluating the spatial integration in Equation (10) produces a set of coupled ordinary equations, which can be solved numerically using the Runge–Kutta method. We implement the first three mode shapes to produce converged and accurate simulation results.

### Experimental characterization

The experimental characterization setup used for testing the device and measuring the initial profile, gap thickness and out-of-plane vibration is depicted in Supplementary Figure S1. The experiment is conducted on a 400-μm microbeam with a lower electrode that spans half of the beam length. This electrode provides an anti-symmetric electrical force to excite the symmetric and anti-symmetric resonance frequencies. The experimental setup consists of a microsystem analyzer, which is a high-frequency laser Doppler vibrometer under which the microbeam is placed to measure the vibration, data acquisition card connected to an amplifier to provide actuation signals of wide range of frequencies and amplitudes, and vacuum chamber, which is equipped with ports to pass the actuation signal and measure the pressure. In addition, the chamber is connected to a vacuum pump that can reduce the pressure to 4 mtorr.

The initial profile of the microbeam is revealed using an optical profilometer. After defining the vertical scanning range and exposure time, a 3D map of the microbeam is generated (Supplementary Figure S2). The combined thickness of the microbeam and air gap is measured to be ~9 μm. In addition, the total length of the microbeam is 400 μm with a fully straight profile and without any curvature or curling.

To characterize the static behavior of the device, we initially biased the microbeam by a slow DC ramp voltage, generated using the data acquisition card, and measured the static deflection. The experimental result is reported in Supplementary Figure S3. The deflection increases until pull in is exhibited at 168 V.

**Figure 3:**
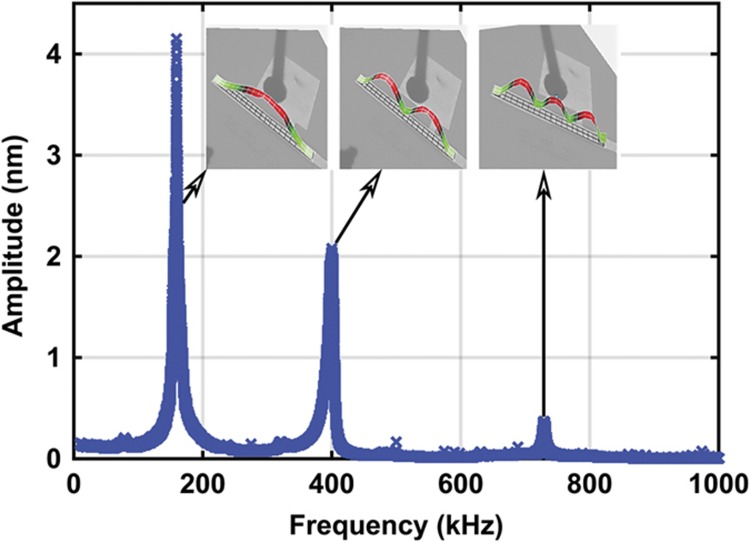
Frequency response curve of the microbeam to a white noise actuation signal with the corresponding mode shape at a load of *V*_DC_=30 V and *V*_AC_=50 V and a pressure of 4 mtorr. The resonance frequencies values are *ω*_1_=160 kHz, *ω*_2_=402 kHz, and *ω*_3_=738 kHz.

We experimentally measured the first three natural frequencies by exciting the device with a white noise signal of *V*_DC_=30 V and *V*_AC_=50 V. The vibration at different points along the beam length is scanned to extract the vibration mode shapes and resonance frequencies. The acquired frequency response curve is depicted in Figure 3, which reveals the values of the first three natural frequencies of *ω*_1_=160 kHz, *ω*_2_=402 kHz, and *ω*_3_=738 kHz. The mode shapes (root mean squared absolute values) are reported in the insets of Figure 3. We observed that all of the points vibrate at *ω*_1_, whereas the mid points are nodal points at *ω*_2_. In addition, at *ω*_3_, there are two nodal points. These results match the clamped–clamped structure’s first, second, and third vibration mode shapes.

## Results

### Frequency response curves

**Figure 4:**
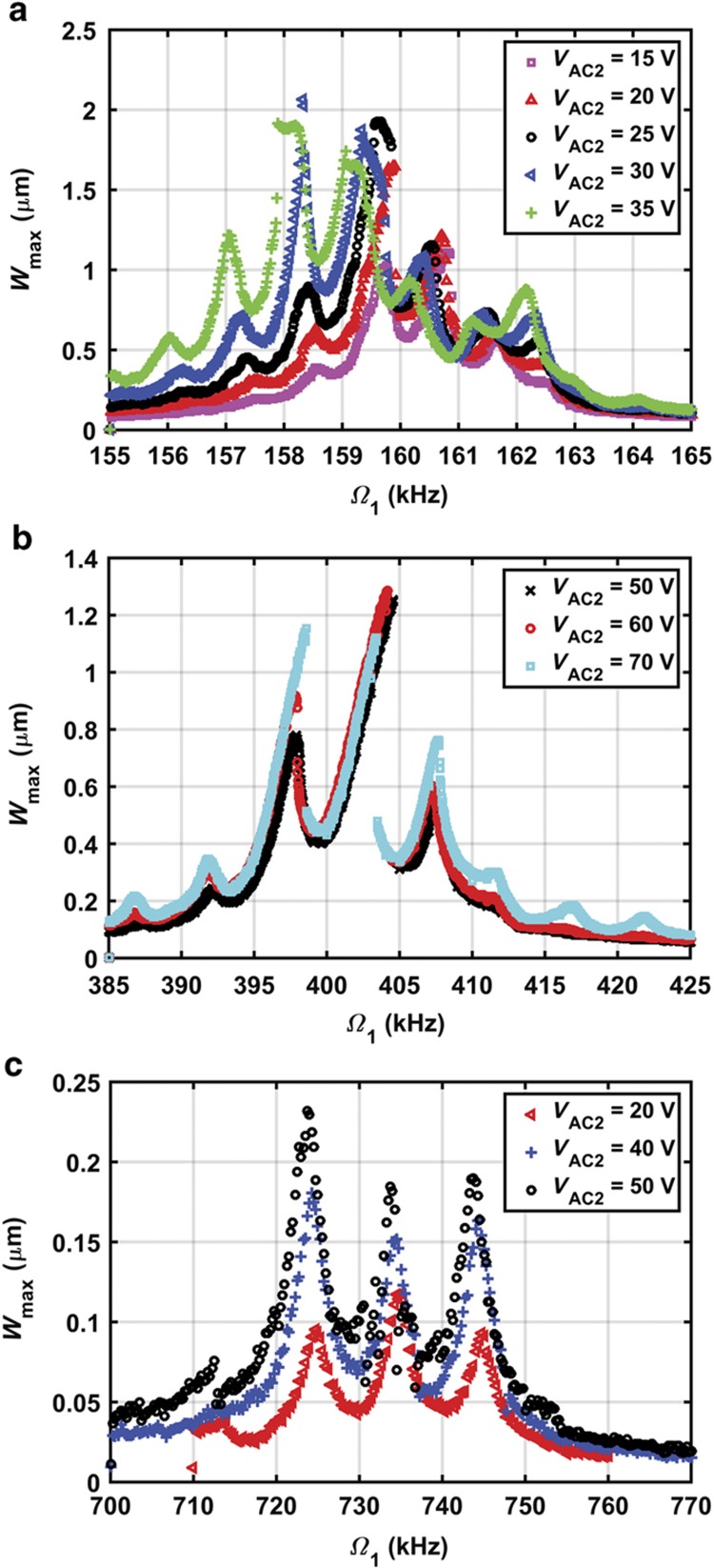
Frequency response curves for different values of *V*_AC2_. (**a**) *V*_DC_=15 V, *V*_AC1_=5 V, and *Ω*_2_=1 kHz near the first mode; (**b**) *V*_DC_=15 V, *V*_AC1_=20 V, and *Ω*_2_=5 kHz near the second mode; and (**c**) *V*_DC_=20 V, *V*_AC1_=40 V, and *Ω*_2_=10 kHz near the third mode.

The nonlinear response of the microbeam is experimentally investigated near the first three modes of vibration. The microbeam is excited using the data acquisition card, and the vibration is detected using the laser Doppler vibrometer. The excitation signal is composed of two AC signals, *V*_AC1_ and *V*_AC2_, superimposed on a DC signal *V*_DC_. The measurements are performed by focusing the laser at the mid-point for the first and third mode measurements and at a quarter of the beam length for the second mode measurements. Then, the frequency response curve is generated by taking the steady-state maximum amplitude of the motion *W*_max_. The generated frequency response curves near the first mode are depicted in Figure 4a. Each curve denotes the frequency response for different values of *V*_AC2_. The results are obtained by sweeping the frequency of the first AC source *Ω*_1_ around the first mode and fixing the second source frequency *Ω*_2_ at 1 kHz. The swept source voltage *V*_AC1_ and the DC voltage are fixed at 5 and 15 V, respectively. The results of sweeping *Ω*_1_ near the second mode while fixing the second source frequency *Ω*_2_ at 5 kHz is depicted in Figure 4b. The swept source voltage *V*_AC1_ and the DC voltage are fixed at 20 and 15 V, respectively. In addition, this experiment is repeated near the third mode, as indicated in Figure 4c, where *Ω*_2_ is fixed at 10 kHz and the actuation voltages *V*_AC1_ and the DC are fixed at 40 and 20 V, respectively. The chamber pressure is fixed at 4 mtorr.

The curves of Figure 4 highlight the effects of *V*_AC2_ on the combination resonances, where new resonance peaks appear at frequencies of the additive type at (*Ω*_1_+*Ω*_2_), (*Ω*_1_+2*Ω*_2_), and (*Ω*_1_+3*Ω*_2_) and the subtractive type at (*Ω*_1_−*Ω*_2_), (*Ω*_1_−2*Ω*_2_), and (*Ω*_1_−3*Ω*_2_)^33^. These resonances appear due to the quadratic nonlinearity of the electrostatic force as well as the cubic nonlinearity caused by mid-plane stretching. It should be noted that in Equation (5), the integral term representing mid-plane stretching indicates *W* and its derivatives to be a positive cubic term, which tends to cause hardening behavior. However, expanding the electrostatic force term in Equation (5) with a Taylor series results in a constant term, that is, representing the static effect, linear term, that is, representing the linear decrease in the natural frequency due to voltage loads, quadratic nonlinearity and other higher order nonlinearities. The strongest nonlinearity is the quadratic one, which is known to cause a softening effect regardless of its sign^17^. In addition, hardening behavior is reported near the first and second resonances. As *V*_AC2_ increases near the first resonance (Figure 4a), the response curves tilt toward the lower frequency values (softening), where the quadratic nonlinearity from the electrostatic force dominates the cubic nonlinearity from mid-plane stretching.

**Figure 5:**
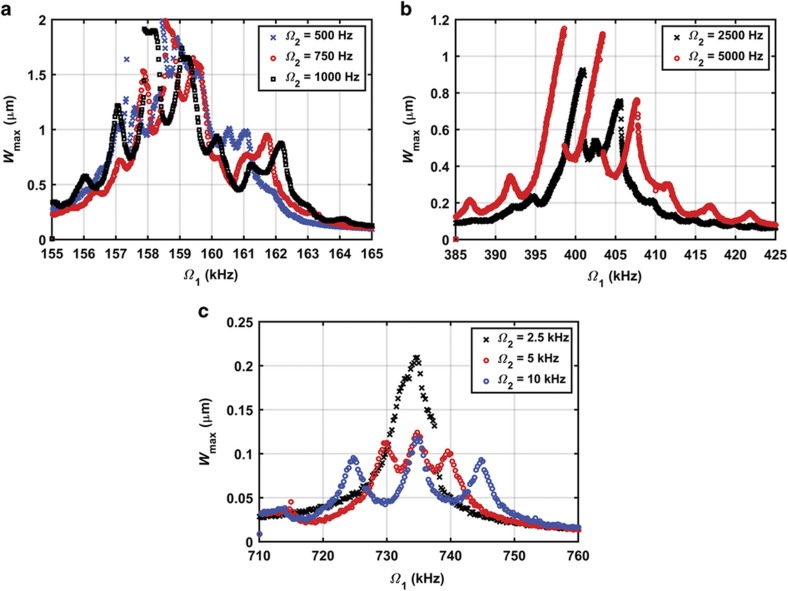
Frequency response curves for different values of *Ω*_2_. (**a**) Near the first mode at *V*_DC_=15 V, *V*_AC1_=5 V, and *V*_AC2_=35 V. (**b**) Near the second mode at *V*_DC_=15 V, *V*_AC1_=20 V, and *V*_AC2_=70 V. (**c**) Near the third mode at *V*_DC_=20 V, *V*_AC1_=40 V, and *V*_AC2_=20 V.

Figures 5a–c reports the results for different values of *Ω*_2_ under the same electrodynamic loading condition near the first, second and third resonance frequencies. As *Ω*_2_ decreases further, a continuous band of high amplitude is formed. This result demonstrates that the multifrequency excitation can be used to broaden the large amplitude response near the main resonance, hence increasing the bandwidth, even for higher order modes.

In addition to the previous results, Supplementary Figure S4 compares the experimentally obtained response owing to a single-frequency excitation of parameters *V*_DC_=15 V and *V*_AC_=5 V to that of a two-source excitation, where another harmonic source of frequency fixed at 1 kHz and amplitude of 10 V is added. The multifrequency response indicates a clear contrast and clear advantage in terms of bandwidth, which can have several practical applications. Typically, resonators of the resonant sensors may not necessarily be driven at the exact sharp peak due to noise, temperature fluctuation and other uncertainties, which results in significant losses and weak signal to noise ratios. The above results prove the ability to control the resonator bandwidth by properly tuning the excitation force frequencies. In addition, by using the partial lower electrode configuration and properly tuning the excitation voltages, the higher order modes of vibration are excited with high amplitudes above the noise level.

### Simulation results

The microbeam dynamical behavior is modeled according to Equation (5) with the unknown parameters *EI*, *N*, and *C*, which are extracted experimentally. All of the results are obtained based on the derived reduced order model. The eigenvalue problem of Equation (9) is solved for different values of the non-dimensional internal axial force *N*_non_ to determine the theoretical frequency ratio *ω*_2_/*ω*_1_ that matches the measured ratio. The theoretical and experimental ratios are matched for *N*_non_=20.82, as reported in Supplementary Figure S5. The axial forces in the surface micromachining process arise due to the residual stress from depositing the different layers of the microbeam at high temperatures and then being cooled down to room temperature. These forces affect the resonance frequency values and their ratio. To extract the flexural rigidity *EI*, we use the static deflection curve and match the theoretical result with the experimental data (Supplementary Figure S3). On the basis of the static solution of Equation (5), we determined that *EI*=0.106×10^−9^ Nm^2^. The damping ratio *ς* is extracted from the frequency response curve of the beam to a single and small AC excitation, where the experimental and theoretical results are matched at a damping ratio *ς*=0.002, as depicted in Supplementary Figure S6.

**Figure 6:**
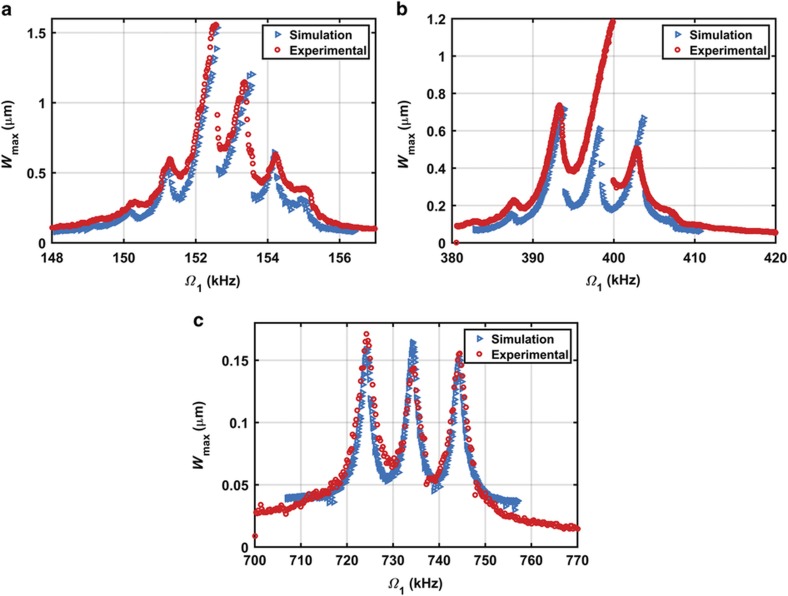
Experimental and simulation results of the microbeam. (**a**) Near the first mode of vibration for *V*_DC_=15 V, *V*_AC1_=5 V, *V*_AC2_=20 V, and *Ω*_2_=1 kHz; (**b**) Near the second mode of vibration for *V*_DC_=20 V, *V*_AC1_=15 V, *V*_AC2_=50 V, and *Ω*_2_=5 kHz; and (**c**) Near the third mode of vibration for *V*_DC_=20 V, *V*_AC1_=40 V, *V*_AC2_=40 V, and *Ω*_2_=10 kHz.

The simulated dynamic response is based on a long-time integration of the modal equations of the reduced-order model of Equation (10) to reach a steady-state response. The first three-mode shapes are used in the reduced-order model to approximate the response. The simulation and experimental results for the multifrequency excitation near the first three modes of vibrations are reported in Figures 6a–c. Using the Galerkin approximation, the model predicts the resonator response accurately near the first and third mode shapes. Near the second mode, long-time integration method failed to capture the complete solution due to the weak basin of attraction near the large response curve, as indicated in Figure 6b. As reported by Batineh and Younis^34^, long-time integration method depends on the size and robustness of the basin of attraction to capture a solution. Another numerical technique needs to be implemented to accurately predict the complete response, such as the shooting technique, which can determine the entire response as well as capture the stable and unstable periodic solutions^12,34^.

## Conclusions

In this report, we investigated the dynamics of an electrically actuated clamped–clamped microbeam excited by two harmonic AC sources with different frequencies superimposed onto a DC voltage near the first three modes of vibrations. After recording the static deflection curve and detecting the first three natural frequencies, a numerical analysis was conducted to extract the device parameters. Then, the governing equation was solved using three mode shapes, which provided a good agreement between the simulation and the experimental results. Moreover, we proved the ability to excite the combination resonance of both the additive and subtractive type. In addition, the ability to broaden and control the bandwidth of the resonator near the higher order modes has been illustrated by properly tuning the frequency of the fixed source. Furthermore, by increasing the fixed frequency source voltage, the vibration amplitude with respect to noise near the higher order modes is enhanced. These capabilities of generating multiple peaks and a wide continuous response band with the ability to control its amplitude and location can have a promising application in increasing the resonator bandwidth for applications such as mechanical logic circuits, energy harvesting and mass sensing.
