# Supplementary material for: Multifrequency excitation of a clamped–clamped microbeam: Analytical and experimental investigation
Source: Microsyst Nanoeng. 2016 Mar 14;2:16002. doi: 10.1038/micronano.2016.2 (PMC6444723; doi:10.1038/micronano.2016.2)
Supplement: Supplementary Information [file micronano20162-s1.pdf]

Supplementary file

Multifrequency excitation of a clamped-clamped microbeam: Analytical and experimental investigation

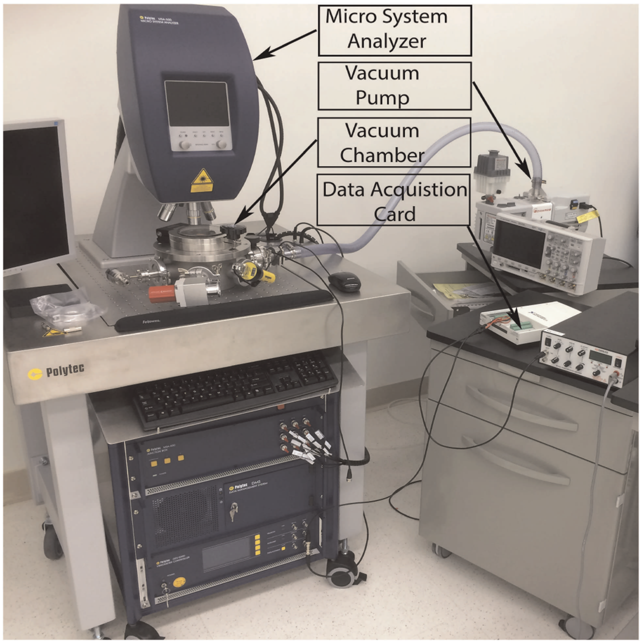

Figure S1 Experimental setup used for testing the MEMS devices.

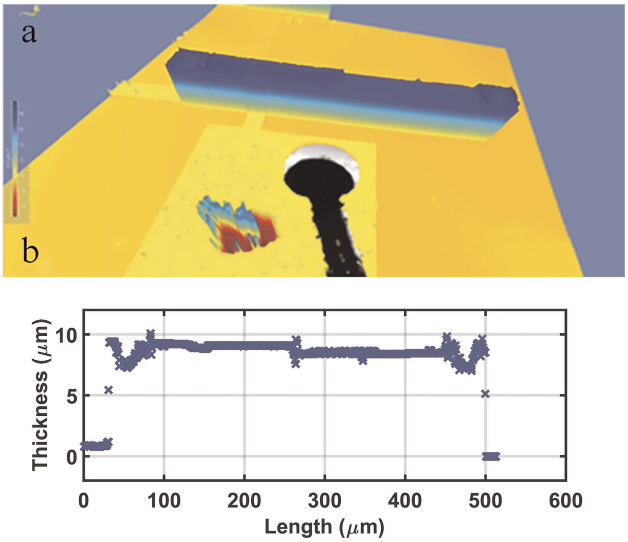

Figure S2 Topography of the microbeam. (**a**) 3D map. (**b**) Cross sectional view of the microbeam profile.

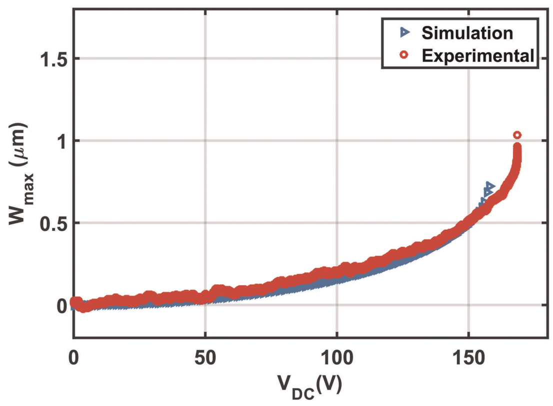

Figure S3 The static deflection of the microbeam.

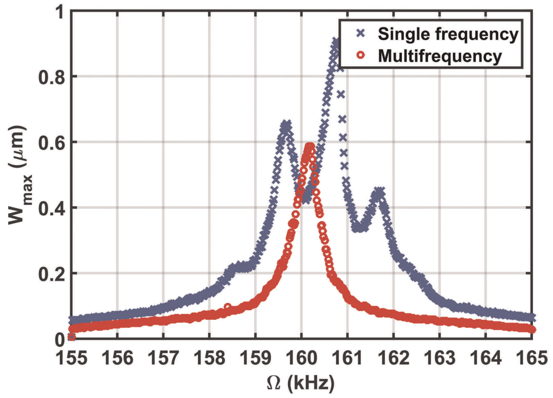

Figure S4 Frequency response curves near the first mode comparing a single frequency excitation response (red circles) at $V_{DC} = 15$ V, $V_{AC} = 5$ V to a multifrequency excitation response (blue triangles) for $V_{DC} = 15$ V, $V_{AC1} = 5$ V, $V_{AC2} = 10$ V and $\Omega_2 = 1$ kHz.

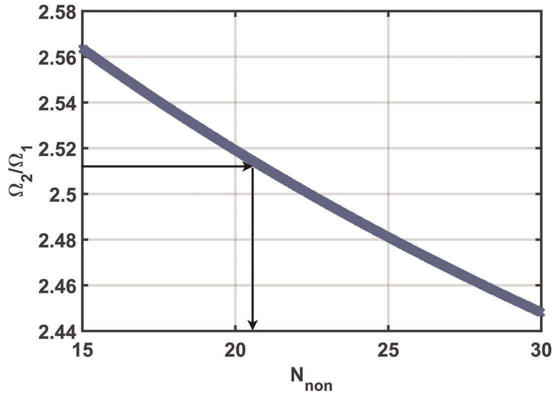

Figure S5 The ratio of the first to second eigen frequencies for different values of the nondimensional axial force N_{non}.

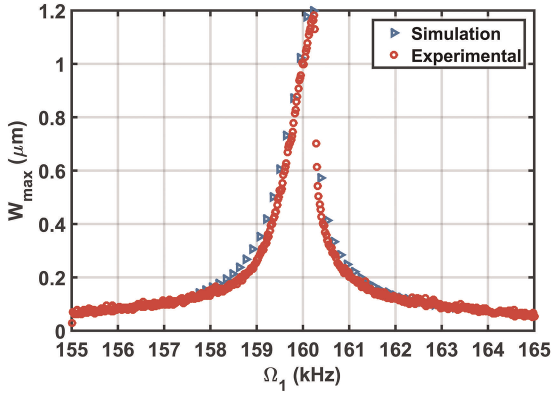

Figure S6 Frequency response curve near the first mode of vibration at $V_{DC} = 25$ V and $V_{AC} = 5$ V.
